# Using Facebook advertising data to describe the socio-economic situation of Syrian refugees in Lebanon

**DOI:** 10.3389/fdata.2022.1033530

**Published:** 2022-11-30

**Authors:** Masoomali Fatehkia, Zinnya del Villar, Till Koebe, Emmanuel Letouzé, Andres Lozano, Roaa Al Feel, Fouad Mrad, Ingmar Weber

**Affiliations:** ^1^Qatar Computing Research Institute, HBKU, Doha, Qatar; ^2^Data-Pop Alliance, New York City, NY, United States; ^3^Freie Universität, Berlin, Germany; ^4^Department of Political and Social Sciences, Universitat Pompeu Fabra, Barcelona, Spain; ^5^UN ESCWA, Beirut, Lebanon; ^6^Department of Computer Science, Saarland University, Saarbrücken, Germany

**Keywords:** Syrian civil war, Lebanon, Facebook advertising data, big data, vulnerability assessment

## Abstract

While the fighting in the Syrian civil war has mostly stopped, an estimated 5.6 million Syrians remain living in neighboring countries^1^. Of these, an estimated 1.5 million are sheltering in Lebanon. Ongoing efforts by organizations such as UNHCR to support the refugee population are often ineffective in reaching those most in need. According to UNHCR's 2019 Vulnerability Assessment of Syrian Refugees Report (VASyR), only 44% of the Syrian refugee families eligible for multipurpose cash assistance were provided with help, as the others were not captured in the data. In this project, we are investigating the use of non-traditional data, derived from Facebook advertising data, for population level vulnerability assessment. In a nutshell, Facebook provides advertisers with an estimate of how many of its users match certain targeting criteria, e.g., how many Facebook users currently living in Beirut are “living abroad,” aged 18–34, speak Arabic, and primarily use an iOS device. We evaluate the use of such audience estimates to describe the spatial variation in the socioeconomic situation of Syrian refugees across Lebanon. Using data from VASyR as ground truth, we find that iOS device usage explains 90% of the out-of-sample variance in poverty across the Lebanese governorates. However, evaluating predictions at a smaller spatial resolution also indicate limits related to sparsity, as Facebook, for privacy reasons, does not provide audience estimates for fewer than 1,000 users. Furthermore, comparing the population distribution by age and gender of Facebook users with that of the Syrian refugees from VASyR suggests an under-representation of Syrian women on the social media platform. This work adds to growing body of literature demonstrating the value of anonymous and aggregate Facebook advertising data for analysing large-scale humanitarian crises and migration events.

## 1. Introduction

Eleven years after the start of the civil war in Syria, more than 5.6 million Syrians have left the country and currently, an estimated 1.5 million Syrian refugees live in Lebanon, nearly a quarter of Lebanon's total population, the largest proportion of refugees worldwide. This massive influx of refugees into the country has overburdened essential services (access to health care, education, water, and sanitation) for both refugees and host communities. Despite national and international efforts to support refugees and host communities, their vulnerability has been exacerbated by additional shocks, such as the pandemic and the current social and economic meltdown marked by the highest inflation rate in the world and the Lebanese currency losing more than 90% of its value since October 2019^2^. Together these factors are pushing Syrian refugees even further into the margins of the Lebanese Society.

In this context, policymakers, development, and humanitarian practitioners should rely on timely and accurate information to plan systems, services, and strategies in the host communities as well as to develop policies aimed at decreasing the vulnerabilities, risks, and challenges faced by both populations. To this end, traditional data sources such as official sources (censuses and surveys) can be expensive, time-consuming and difficult to collect, resulting in small samples with little geographical disaggregation. Also, official statistics are updated over longer periods of time which can make the information they contain outdated, especially within the highly volatile political and socioeconomic context of the Arab region.

On the other hand, in order to improve timeliness and frequency, non-traditional data sources such as call detail records (CDR), the Global Database of Events Language, and Tone (GDELT project^3^.) and Earth Observation data (EO) among others, are presented as an alternative solution that can range from the creation of official statistics (Letouzé and Jütting, 2015; Alliance, 2016) and as a complement to existing data and research approaches into sustainable development (Weber et al., 2021). These non-traditional data sources as well as the techniques used to process them have shown innovative advances in recent years in some thematic areas such as migration and mobility (Mazzoli et al., 2020; Luca et al., 2022), violence and crime (Bogomolov et al., 2016), and gender inequalities (Kashyap et al., 2020) are just a few examples.

To enhance the use of non-traditional data sources, this work highlights the use of online advertising data to improve official statistics with subnational and gender-disaggregated statistics. Specifically, we used advertising data from Facebook extracted from Facebook's Adverts Manager tool to estimate income- and poverty- related indicators on the level of regions in Lebanon. Facebook Adverts Manager was developed to attract more advertisements and improve the accuracy of audience targeting, which allows advertisers to give detailed specifications of the type of users to be targeted with ads through the targeting of dimensions that range from information explicitly reported by Facebook users such as gender, education, current location, and home location, to information automatically inferred from their interactions on Facebook and affiliate websites^4^). Facebook also allows marketers to target migrants living within a given country, for example people born in one country living abroad, or migrants living in a specific country (Rampazzo et al., 2021; Vieira et al., 2022). Estimates on how many Facebook users match these criteria can be obtained free of charge through the Adverts Manager and has already been used to monitor the flow of refugees and migrants (Palotti et al., 2020).

Knowing that data published in Meta's advertising resources indicates that Facebook had 3.15 million users in Lebanon in early 2022 which corresponds to 59.2% of the eligible population, that is, people aged 13 and above^5^, this work used Facebook advertising data along with UNHCR's Vulnerability Assessment of Syrian Refugees in Lebanon (VASyR 2019), to describe the spatial variation in socioeconomic characteristics of Syrian refugees. The document starts by detailing user attributes from Facebook users through advertising data. Then, model results are presented. Finally, the discussion section concludes the paper, by reflecting on the usefulness of the proof of concept.

## 2. Materials and methods

### 2.1. Facebook advertising audience estimates

As a social media platform, Facebook generates revenues from advertising. The Facebook advertising platform provides advertisers with a wide range of targeting options. These targeting options include geographic location, demographics such as age and gender, self-declared education levels, inferred user behavior such as devices and network connection types used to access the platform, language as well as interests among others. For a specified set of targeting criteria, before launching the ad, the advertiser is provided with an estimate of Monthly Active Users (MAU) matching these criteria. For example, Facebook estimated there were 160,000 female users aged 13+ from the South Governorate of Lebanon at the time of data collection. These audience estimates can then be used for campaign planning and budgeting purposes by advertisers.

Beyond advertising purposes, Facebook's ad audience estimates are a valuable source of information on the Facebook user population across various locations and can be a useful source of data for research purposes. Prior studies have utilized Facebook's advertising audience estimates to investigate a variety of topics such as analyzing population health (Araujo et al., 2017), measuring digital gender inequalities (Fatehkia et al., 2018; Garcia et al., 2018) as well as mapping subnational socioeconomic indicators (Fatehkia et al., 2020). Various studies have investigated the utility of such data to estimate the size of migrant populations (Zagheni et al., 2017) and to quantify global migration patterns (Spyratos et al., 2019).

In addition to studying general migration patterns, Facebook ad data has also been used for studying crisis-based migration. Alexander et al. (2019) use these data to study the migration pattern of Puerto Ricans to mainland USA in the aftermath of hurricane Maria. In the context of refugee crises, Palotti et al. (2020) use these data to estimate the spatial distribution of Venezuelan refugees at national and subnational resolutions in neighboring Latin American countries. More recently, Minora et al. (2022) use these advertising audience estimates to study the migration of Ukraine refugees to EU countries while Leasure et al. (2022) use these data to quantify internally displaced populations within Ukraine. While the aforementioned studies rely on passively collected data from advertising audience estimates, there is growing body of work that uses data collected actively through surveys administered by advertising on social media platforms such as Facebook (Pötzschke and Braun, 2017; Pötzschke, 2022).

#### 2.1.1. Data collection

In addition to its online advertising platform, Facebook also provides an Application Programming Interface (API) that can be used to create and manage advertising campaigns programmatically using automated scripts. The advertising audience estimates can also be collected using Facebook's marketing API^6^. For this study we collected data through the marketing API using the pySocialWatcher library^7^, a wrapper library in the python programming language which automatically collects these data through Facebook's marketing API for a specified set of targeting criteria. The data collection was done in November 2019. The following sections provide more information about the data collected.

##### 2.1.1.1. Geographic locations

**Figure 1:**
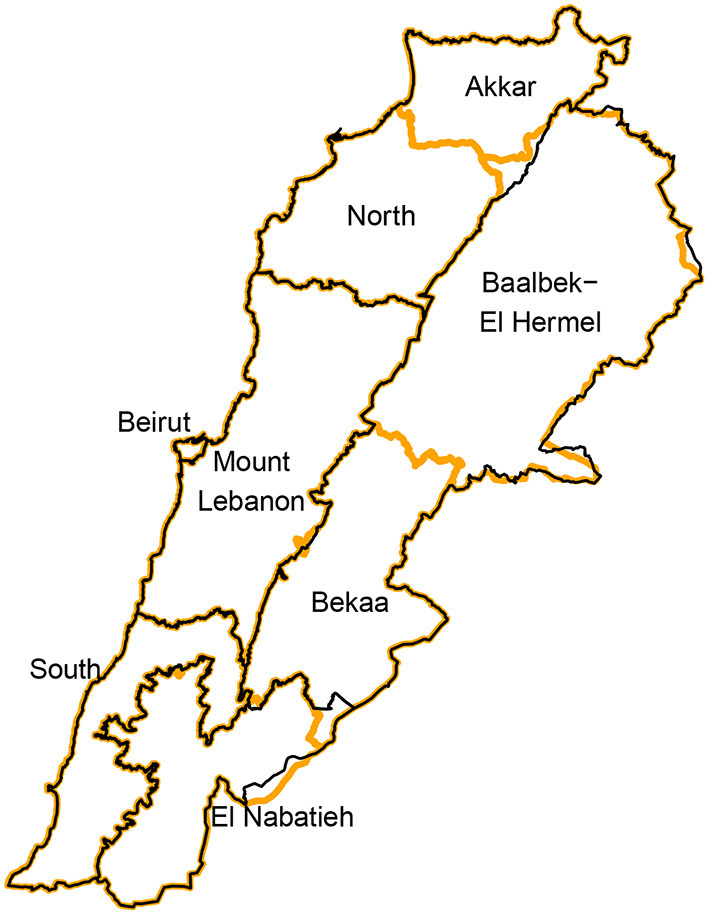
Governorates of Lebanon (orange) overlaid with Facebook regions (black) that can be targeted for advertising.

Data was collected at the national, administrative region, and city levels. Subnational data is used to test the potential of Facebook Data to provide insights at higher spatial resolutions. Facebook regions typically match the first-level subnational administrative division within a country. In Lebanon, the first-level administrative division are the governorates (Muhafazah). At the time of data collection, Lebanon was divided into eight governorates which are shown and labeled on the map in Figure 1 (orange boundaries). The figure also displays the six regions that could be targeted through Facebook advertising (black boundaries). As can be seen in the figure, four of the eight governorates within Lebanon could be directly targeted as regions through the Facebook advertising platform. For the remaining four governorates, it was not possible to collect Facebook advertising data separately because the platform had grouped these four governorates under two regions, reflecting an older administrative division of the country. In particular, the Akkar and North governorates could only be targeted as one region on Facebook (the North region); likewise for the Bekaa and Baalbek-El Hermel governorates which were grouped under the Bekaa region on the Facebook advertising platform. In our analysis we present results for these six regions for which advertising audience estimates data is available.

In order to test the potential of using such data to gain insights at smaller spatial resolutions, we also collected audience estimates data at the city level. At the time of data collection, the Facebook advertising platform provided a list of 770 cities that could be targeted in Lebanon. It should be noted that the city geographic area is a categorization provided by the Facebook advertising platform and cities presented by Facebook may correspond to cities or other similar administrative divisions in the country. Unlike regions, no exact geographic boundaries are provided for cities; instead, the platform provides a pair of latitude and longitude coordinates denoting the city location. Looking at the geographic locations and names of cities on Facebook alongside the administrative divisions of Lebanon suggests that in some cases the cities may correspond to cadastres of Lebanon while in other cases they correspond to larger cities consisting of multiple cadastres. However, due to the lack of precise geographic boundaries, the exact matching between cities and administrative divisions remains unclear. Another challenge with the city level data is the high level of sparsity whereby many places have few or no users. For privacy reasons, Facebook's API returns an estimated audience size of 1,000 for locations with 1,000 or fewer users. Out of the 770 cities, 195 (25%) had a total estimated user count of more than 1,000. When looking at the refugee population, as defined in Section 2.1.1.3, the number of cities with more than 1,000 users reduces further to 49 (6.4%). Due to these limitations, we do not present any city level analysis here.

##### 2.1.1.2. User attributes

The Facebook advertising platform provides a wide array of targeting options based on a variety of self-declared and inferred user attributes. For this study, we collected data for various combinations of the following user attributes:

- **Age:** data was collected for all users (ages 13+) as well as for smaller age groups in 5-year age buckets (15–19, 20–24, …, 60–64) and the 65+ age group.
- **Gender:** we collect data for (i) all users, (ii) Male, and (iii) Female users.
- **Language:** this attribute targets users based on language. We collected data for (i) all users (no language targeting specified) and (ii) Arabic speakers.
- **Migrant status:** Users can be targeted based on their migrant status, referred to as expat status by Facebook, defined as users living outside of their home country based on Facebook algorithms. It is possible to target all expats (regardless of country of origin) as well as expats from specific countries of origin, i.e., expats from country X defined as users who used to live in country X who now live abroad. A total of 89 countries of origin were supported at the time of data collection. For this attribute we collected data for (i) all users (no expat targeting specified), (ii) users who are expats (all expats), (iii) all expats excluding expats who are from specific Arabic-speaking countries of origin (these are expats from countries for which an “expat from country X” targeting category exists, namely, Algeria, Jordan, Kuwait, Lebanon, Morocco, Qatar, Saudi Arabia, and UAE.), and (iv) users who are not expats.
- **Self-declared education:** Facebook users can provide information about their education level on their profile. This attribute allows for targeting users based on their self-declared education levels. We collected data for (i) all users (no education targeting specified), (ii) unspecified education level (users with unknown/unspecified educational status), (iii) users with a high-school degree, (iv) users with a college degree and above (including Masters/Doctorate), and (v) only users with Masters/Doctorate.
- **Device/Network connection types:** Ads can be targeted to users based on the network connections and device types they use to access Facebook. We collected data for users with the following network connections and device types: (i) 2G Network, (ii) 3G Network, (iii) 4G Network, (iv) WiFi, (v) iOS Mobile OS, (vi) Android Mobile OS, (vii) High-end phone (defined as users with a range of high-end phones that were available for targeting at the time of data collection. These devices were Apple iPhone X, 8 and 8+ as well as Samsung Galaxy S8, S8+, S9, S9+).

##### 2.1.1.3. Defining host and refugee communities

As the focus of this study is to provide estimates and insights on Syrian refugee populations in Lebanon, we attempted to define this user population in the Facebook advertising data using a combination of different targeting criteria. A natural choice for defining migrants from a given country of origin on Facebook would be to use the “Expats from X” targeting criteria which would define users who used to live in country X but now live abroad. However, such a targeting category is not available for users from Syria as a country of origin. As it was not possible to define the Syrian refugee population this way, we used a combination of expat status and language to define the Syrian refugee population of Facebook users. We define the Syrian refugee community as Facebook users who speak Arabic and who are expats [excluding expats from Arabic-speaking countries of origin that can be targeted on Facebook; see point (iii) under the “Migrant status” bullet point in the previous section]. We exclude expats from other Arabic-speaking countries of origin in order to narrow the user demographic to Syrian refugees as much as possible.

A point worth considering at this point is the distinction between expats and refugees. We use the estimates of expats on Facebook, defined as users living outside of their home country as determined by Facebook's algorithms, to characterize the population of interest, i.e., Syrian refugees. While expats and refugees are indeed distinct concepts, in the context of Lebanon, it is not as easy to distinguish Syrian expats vs. refugees. To our knowledge, there are no disaggregated population statistics on the population of Syrians in Lebanon based on their expat or refugee status. Nevertheless, given the large share of Syrian refugees in Lebanon, representing nearly a quarter of the total population, it is likely that a significant share of Syrians in Lebanon are refugees. Hence, it seems plausible that a considerable proportion of Arabic speaking expats on Facebook, excluding other countries, are likely to be Syrian Refugees. While bearing in mind this distinction, we use the term refugee community to refer to this group in our analysis.

For the purposes of analysis and comparison, we also define the Facebook user groups who are likely to be the Lebanese host population. More specifically, we define the host population as users who are not expats. We use these definitions whenever referring to host and refugee users/community in the results.

### 2.2. Survey data

In order to explore the potential and limitations of the Facebook advertising audience estimates data, we validate it against available ground truth data sources. The estimates of refugee user populations were compared against UNHCR registration figures for Syrian refugees in Lebanon (UNHCR, 2019b)^8^. Data on the age, gender, and socioeconomic status of Syrian refugees in Lebanon came from the 2019 Vulnerability Assessment of Syrian Refugees (VASyR) (UNHCR et al., 2019)^9^ data and reports.

As explained in earlier sections, we analyze six regions in Lebanon based on the regions available from the Facebook advertising data. Therefore, the ground truth data for Akkar/North and Bekaa/Baalbek-El Hermel governorates were aggregated in order to match the Facebook regions. The counts of registered Syrian refugees were summed up while income/poverty figures were averaged. The resulting averaged poverty/income figures were very similar regardless of whether we used a non-weighted arithmetic average or a population-weighted average that weighted based on the number of registered Syrian refugees in each governorate; the calculated poverty figures were within 1% point of each other while for the income figures the difference was less than two USD. As both approaches resulted in similar figures, and because the registration figures of Syrian refugees may not reflect the population changes since 2015 when new registrations were suspended, we used the non-weighted average to aggregate poverty/income for these governorates.

## 3. Results

### 3.1. Demographics

At the country-level, there were 2.6 million Facebook users from the host community and 720,000 users from the refugee community in Lebanon. According to UNHCR (2019b), there were 916,113 registered Syrian refugees in Lebanon as of November 2019, while the government of Lebanon estimated 1.5 million Syrian refugees (UNHCR, 2019a) in the country. It should be noted that UNHCR suspended new registrations for Syrian refugees at the beginning of 2015 as per the instructions of the Lebanese government (UNHCR, 2021). As a result, the number of registered Syrians refugees has been in gradual decline since then.

**Figure 2:**
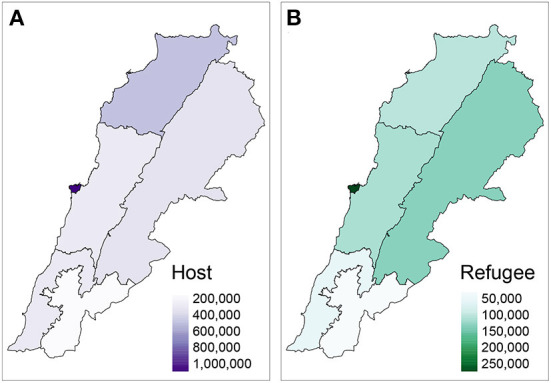
Distribution of Facebook users across different regions of Lebanon for the host **(A)** and refugee **(B)** communities.

Looking subnationally, Figure 2 shows the distribution of Monthly Active Facebook Users from the host and refugee communities across different governorates of Lebanon. As can be seen in Figure 2A the top 3 regions with the highest numbers of users from the host community were Beirut, North, and Bekaa, respectively. Figure 2B shows the distribution of users for the refugee community. Beirut is the region with the highest population of users from the refugee community followed by Bekaa and Mount Lebanon.

**Figure 3:**
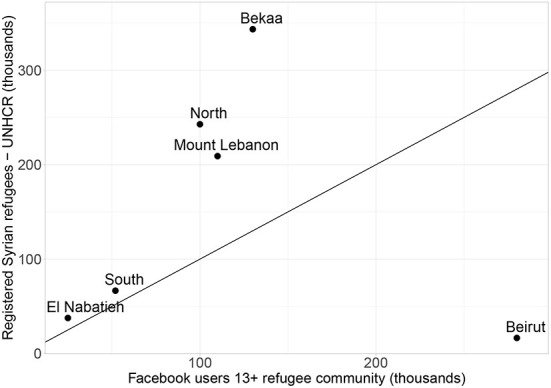
Scatter plot of the number of Facebook users aged 13+ (refugee community) against the number of registered Syrian refugees based on UNHCR data at the governorate level. The plotted line is the diagonal line where the number of users equals the number of registered refugees.

Figure 3 plots the number of users from the refugee community in each region against the number of registered Syrian refugees based on UNHCR data. As can be seen in the plot, regions with greater numbers of registered Syrian refugees also have a larger number of Facebook users from the refugee community. However, the Beirut governorate is an outlier to this observed trend as the number of Facebook users from the refugee community in Beirut is much larger than the registration figures. A possible explanation for that might be that a significant number of Syrian refugees comes to Beirut as opportunities to work are more plentiful. While registration is easier in other regions, where they leave family members behind due to the lower cost of living outside of Beirut. An additional or alternative explanation could lie in noise and inconsistencies in Facebook's geolocation for the Beirut region. When looking at smaller custom locations within the Beirut region (using Facebook's custom locations one can target a specified radius around a given geographic coordinate), the provided user estimate sometimes varies by large amounts for nearby locations. As an example, an estimated 2,400 users live in the 1 mile radius around (latitude:33.8898, longitude:35.4805) while the user estimate is 695,000 for the 1 mile radius around (latitude:33.8902, longitude:35.4805) which is 44 m apart from the previous location. However, in general, we do not observe such geolocation issues for other places. Based on this observation, it seems plausible that issues with Facebook's geolocation within the region could result in overestimation of the user population in Beirut.

### 3.2. Mapping income/poverty

Information on the socioeconomic situation of refugee populations is crucial for policy making and planning interventions. In their work mapping sub-regional wealth for low and middle income countries using a combination of different data sources, including connectivity data from Facebook, Chi et al. (2022) discovered that features related to mobile connectivity were highly predictive of wealth and appeared among the topmost important features in their model. In this section, we show how data on the network connections and device types of Facebook users can be used to estimate income and poverty among the refugee population in the governorates of Lebanon.

**Figure 4:**
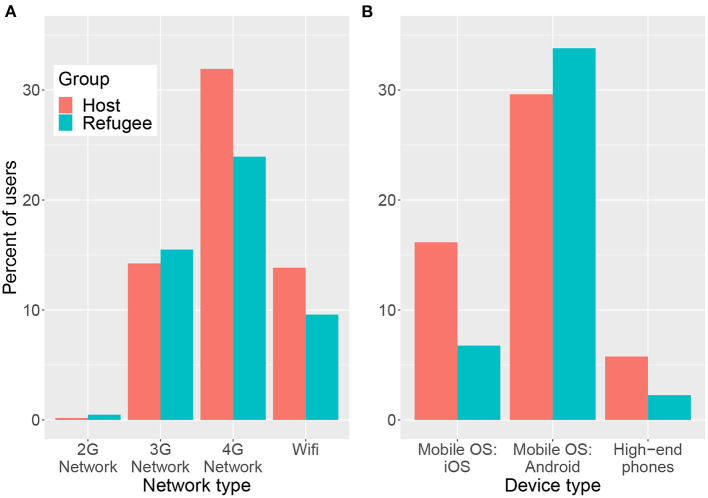
Percentage of Facebook users aged 13+ from the host and refugee communities in Lebanon using various network connections **(A)** and device types **(B)** to access the platform. Note that the percentages across the different network connections or device types may not add to 100% as users may be using other means to access Facebook such as a cable internet connection, other mobile operating systems or a desktop computer.

Figure 4 shows the percentage of users from the host and refugee communities in Lebanon who use various network connections (Figure 4A) and device types (Figure 4B) to access Facebook. The plot reveals differences between the two population groups in the types of devices and connection types that they use. Compared to the host population, a larger percentage of refugees use lower-end 2G and 3G network connections while 4G and WiFi are more commonly used by the host population. Similar patterns can be observed for the devices and mobile Operating Systems (OS) used. The host population use more iOS devices and higher-end phones while the refugees use more Android devices which generally tend to have a lower cost. These observations hint at the socioeconomic gaps between the host and refugee populations, suggesting that refugees are more socioeconomically disadvantaged than the host population.

Table 1 shows the Pearson correlation between the percentage of refugee users with various devices/network connections and the governorate level income and poverty levels for Syrian refugees based on VASyR surveys. Among the variables presented in the table, the percentage of users with an iOS device is the most strongly correlated with both income and poverty, followed by the percentage of users with High-end phones. While the number of data points, based on six governorates, is too small to draw statistical conclusions, the strong spatial correlation observed is in line with previous work (Fatehkia et al., 2020) that used device types on Facebook, including iOS devices, to estimate subnational measures of wealth in India and the Philippines. In another study, Palotti et al. (2020) observed strong correlations between iOS device usage on Facebook and country-level GDP per capita for countries in South America; when applying their model using the percentage of iOS device users to estimate the socioeconomic situations of likely-to-be Venezuelan migrants (for which there was no ground truth data available) they observed plausible general patterns with people in rural areas of neighboring countries being worst off and people who could afford to get on a plane and travel further away (to the US or Spain) being best off.

**Table 1:** Pearson correlation between the percentage of refugee users with various network/device types and income/poverty from VASyr 2019 based on data for the six governorates of Lebanon.

**Device**	**Perc. below poverty line**	**Per capita income**
2G network	0.396 (*p* = 0.438)	−0.085 (*p* = 0.873)
3G network	−0.271 (*p* = 0.604)	0.572 (*p* = 0.236)
4G network	−0.219 (*p* = 0.677)	0.534 (*p* = 0.275)
Wifi	−0.856 (*p* = 0.029)	0.789 (*p* = 0.062)
iOS OS	−0.968 (*p* = 0.002)	0.951 (*p* = 0.004)
Android OS	−0.557 (*p* = 0.250)	0.671 (*p* = 0.145)
High-end phones	−0.913 (*p* = 0.011)	0.845 (*p* = 0.034)

Correlation *p*-values are provided in brackets.

**Figure 5:**
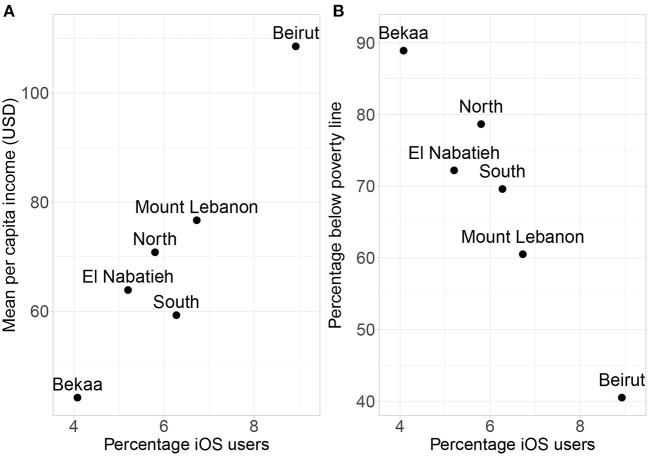
Scatter plots of the percentage of Facebook users from the refugee community who use an iOS device against mean per capita income **(A)** and percentage below poverty line **(B)**.

Figures 5A,B show a scatter plot of the percentage of iOS users among refugees on Facebook and income and poverty levels respectively for the governorates of Lebanon. To test the predictive performance of iOS device usage we fit a single-variable regression model which is then evaluated in a Leave-One-Out Cross Validation (LOOCV) setting as follows. We fit the model on 5 out of 6 regions and use it to predict for the left-out region; this is then repeated again, each time leaving out one region and using the model fitted on the other regions to make a prediction for the left-out region. Based on the cross-validated predictions, the single-variable model with iOS devices as a feature achieves an R-squared of 0.895 (predicting percentage below poverty line) and 0.801 (predicting per capita income).

Consistent with prior work, the analysis here suggests that the device types of Facebook users can be used to estimate the socioeconomic situation of Syrian refugee population at the subnational level in Lebanon. However, it is difficult to say whether these measures distinguish the vulnerability of the refugee population in particular from that of the host or the overall population. While we were not able to find ground truth measures of the poverty/income levels among the host population for the governorates of Lebanon to compare with the refugees, we observe strong spatial correlations (Pearson cor. 0.963, *p* = 0.002) between the percentage of iOS users on Facebook from the host and refugee populations. This suggests that the vulnerability of the refugee population is likely to be similar to that of the general population, i.e., governorates where the host population is generally poor are also places where the refugee population is poor. Nevertheless, the percentage of iOS users from the refugee population are slightly more strongly correlated with the poverty/income of the refugee population than the percentage of iOS users of the host population (Pearson cor. between the percentage of iOS users from the host population and percentage of Syrian refugees below poverty line is −0.956, *p* = 0.003, and with per capita income it is 0.930, *p* = 0.007). These observations suggest that using the device type of refugee users on Facebook provide a lightly better estimate of poverty/income levels of the refugee population than using the device type usage of the host population. This provides some weak evidence that using data about the refugee population on Facebook does somewhat better at capturing the population vulnerability of the refugees than using data from the host population.

#### 3.2.1. Trends across time

Table 2 reports the percentage of iOS device users across the governorates of Lebanon for two time periods of November 2019 and July 2020. As can be seen from the table, for both user groups, there was a slight increase in the percentage of iOS users for almost all governorates. Simply applying the regression model fitted on the 2019 data to make predictions from the 2020 data would therefore result in predicting a slight increase in income levels and a drop in poverty levels in Lebanon. However, based on the 2020 VASyR report (UNHCR et al., 2020) the COVID-19 pandemic plunged many refugee households into poverty with the percentage in extreme poverty increasing from 55% in 2019 to 89% in 2020.

**Table 2:** The percentage of iOS users for the host and refugee Facebook users in 2019 and 2020 for Lebanon and its governorates.

	**Host**		**Refugee**	
**Location**	**Nov 2019**	**Jul 2020**	**Nov 2019**	**Jul 2020**
Lebanon	16.15	17.31	6.76	7.35
Beirut	20	21.82	8.93	10.83
Bekaa	11.52	11.67	4.08	4.06
Mount Lebanon	16.21	15.22	6.73	6.52
El Nabatieh	12	13.08	5.20	6
North	12.86	13.40	5.80	6.36
South	13.67	14.12	6.27	7.02

While an increase in the percentage of iOS users may be indicative of increased income levels, there may be other non-income factors that contributed to the observed increase in usage of iOS devices. One potential explanation is an increase in the market share of iOS devices due to the introduction of more budget-friendly product lines of phones (such as iPhone SE) by Apple^10^. Another possibility is that older iOS devices are recirculated at lower prices in secondary markets as newer models are introduced. In such a case, looking at the percentage of users who own the latest, higher-end newly-released iOS phone models across different years may be a stronger indicator of changes in income levels over time.

Based on these observations, using a regression model fitted on data from 1 year may not generalize across time for estimating the absolute levels of poverty/income for future years as the percentage of device users may change due to other non-income related factors. However, the observed relationship between the percentage of iOS device users and income/poverty levels continues to hold for the 2020 data [Pearson cor. with poverty: −0.929 (*p* = 0.007) and cor. with income: 0.846 (*p* = 0.034)]. So, while the regression model specifying the relationship between percentage of iOS users and poverty/income levels might change over time, the observed correlations appear to be more robust across the two time periods. For many applications where the aim is to allocate resources to those most in need, identifying the most vulnerable regions (or in other words, the relative ranking of the different locations) may be more relevant than estimating the absolute levels of income or poverty in each location. Hence, the percentage of iOS device users is useful for identifying the more vulnerable regions even though the exact linear relationship with poverty/income may need to be re-calibrated for different time periods.

### 3.3. Disaggregating the results

#### 3.3.1. Population pyramid

**Figure 6:**
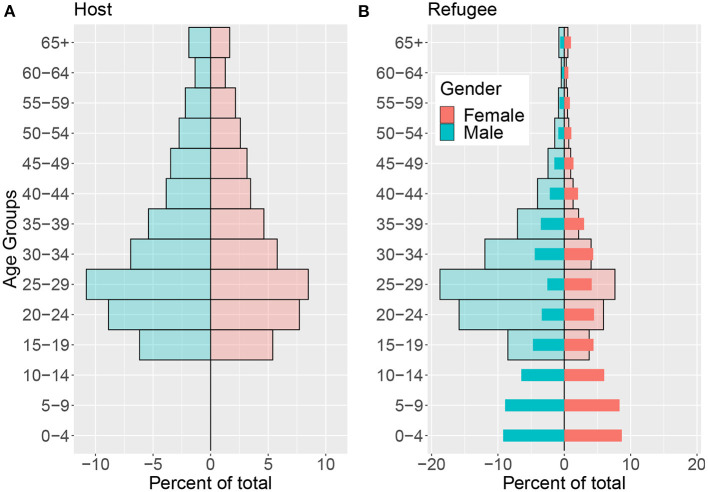
Population pyramids. **(A)** Shows the population distribution by age and gender of Facebook users aged 13+ for the host community. **(B)** Shows the age and gender distribution of Facebook users 13+ for the refugee community (translucent bars) overlaid with the refugee population pyramid from survey data (thin, solid bars).

Figure 6A shows the population pyramid of Facebook users from the host community in Lebanon for 5-year age groups. Although there are overall more male users in the host Facebook user population, the population is roughly gender-balanced with a large young population. Figure 6B shows the population pyramid for Facebook users from the refugee community (translucent bars) overlaid with the population pyramid of Syrian Refugees based on VASyR surveys. In contrast to the host community, women are under-represented in the refugee Facebook user population with the number of men being more than twice the number of women.

These observations highlight the importance of considering user demographics when working with social media data, as some user groups may be over or under represented in the data. In the case of the Syrian refugees, younger men are over-represented while women are under-represented. Moreover, given the minimum age requirements for opening a Facebook account, children are not represented on the platform.

#### 3.3.2. Education

In this section, we explore insights on the educational levels of Syrian refugees in Lebanon that can be derived from self-declared education levels of Facebook users.

**Figure 7:**
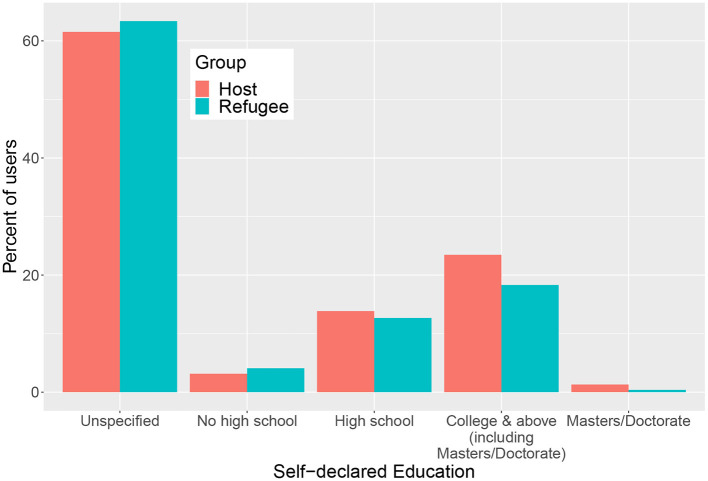
Percentage of users in Lebanon aged 13+ with various self-declared education levels for the host and refugee users.

Figure 7 shows the fraction of users from the host and refugee communities with various self-declared education levels on Facebook. As can be seen in the plot, majority of users (a little over 60%) from both groups do not declare an education level on their profile. Refugee users generally tend to have lower education levels compared to the host population as evidenced by the slightly higher percentage of refugee users with no high school degree compared to the host population and the smaller percentage of the refugee population (18.3%) with a college degree as compared to the host population (23.5%). According to the Lebanese Republic Central Administration of Statistics (CAS) et al. (2020) survey for the year 2018–2019, 25% of the Lebanese population and 6.3% of the non-Lebanese population have a university or higher degree. Compared to these survey data, the percentage of the refugee group on Facebook with a college degree is about three times higher than that of the non-Lebanese population suggesting that users with a higher education level are likely to be over-represented in the sample of refugee Facebook users.

**Figure 8:**
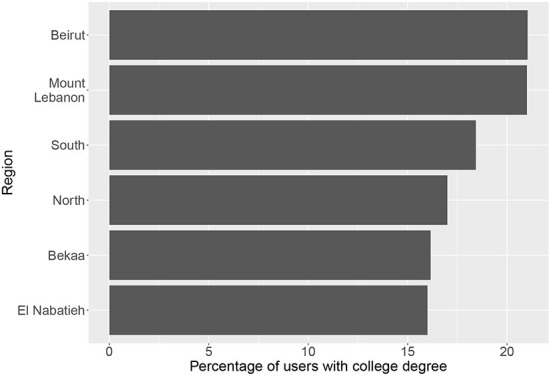
Percentage of Facebook users from the refugee community aged 13+ with self-declared college education across different governorates of Lebanon.

Looking at the subnational level, Figure 8 shows a bar plot of the fraction of refugee users with a self-declared college degree across the governorates of Lebanon. Beirut and Mount Lebanon have the highest proportion of college graduates among the refugee community with about 21% of users in these governorates having a college degree. El Nabatieh and Bekaa, on the other hand, have the lowest number of college graduates across all governorates with about 16% of refugee users having a college degree.

Beyond the information on education levels of host and refugee communities on Facebook, the analysis here reveals insights into potential biases in the sample of Facebook users. As mentioned earlier, college-educated users are likely to be over-represented, particularly among the refugee Facebook users. Another source of potential bias is the fact that the majority of users does not declare an education level. If the tendency to declare an education level is affected by the user's educational attainment, then this can result in some education levels being over or under represented in the data. This highlights the importance of validating social media data against other available data sources such as surveys and keeping in mind potential biases when interpreting results.

#### 3.3.3. Demographically disaggregated vulnerability

Beyond aggregated estimates of population vulnerability, having demographically disaggregated vulnerability figures for different segments of the population (such as by gender) can enable targeted humanitarian assistance to those most in need. Social media advertising data can be useful in this regard due to the availability of demographically disaggregated user estimates by various attributes such as gender, age, and education level. In their work mapping subnational socioeconomic indicators in India and the Philippines using device types on Facebook, Fatehkia et al. (2020) explored the use of gender disaggregated device/network types on Facebook to make estimates separately for men and women. Their models using iOS devices, predicted higher levels of wealth for women compared to men especially in India where the gender disparity on Facebook was larger, making the predictions implausible. This was in line with earlier findings by Magno and Weber (2014) who observed that in locations with greater gender disparities, women had a higher online status than men as defined by a variety of measures.

As can be seen from the aforementioned studies, biases in the distribution of users on the social media platform will influence any demographically disaggregated estimates. In locations where fewer women are online and on Facebook compared to men, it is expected that the sample of women on the platform are “exceptional” in some sense as they may come from higher socioeconomic backgrounds and have greater access to higher end device types. The same applies to disaggregation by other user demographics. It is therefore crucial to be aware of such biases and to attempt to correct for them. Fatehkia et al. (2020), for example, observed that using the gender-disaggregated Facebook penetration as a variable resulted in more plausible estimates of wealth by gender.

In this section, we have attempted to highlight some of the demographic biases in the sample of Facebook users in Lebanon. Provided that the observed biases are constant spatially, across different regions of Lebanon, they will not affect the aggregated estimates of population vulnerability. However, if the aim is to disaggregate such vulnerability estimates by demographics, it will be important to correct for such biases by, for example, using the Facebook penetration. Due to the lack of reliable ground truth population estimates in Lebanon, we did not explore the use of Facebook penetration as a variable. Further, due to sparsity considerations, whereby the pool of available users shrinks as we disaggregate the population by region, expat status, language, device type, and other demographic attributes, we were not able to explore demographically disaggregated vulnerability estimates. However, this could be interesting to explore in other contexts where sufficient data is available for the different demographic subgroups.

## 4. Discussion

This study has shown that Facebook advertising data can help capture geolocated socioeconomic information on the population of Syrian refugees in Lebanon. Facebook-derived indicators such as the network connection or the type of mobile operating system used have been shown to be highly predictive for estimating poverty and income on the subnational level of Lebanese governorates and along other dimensions of disaggregation such as age groups and educational attainment. Even though this analysis is based on a very small dataset from six governorates, the observed correlations are significant at a 5% level, and are consistent with results from earlier studies using Facebook Ads Manager data for poverty assessments. In an attempt to validate these observations at a more fine-grained spatial resolution, we applied our analysis on city-level data from the Facebook advertising platform. Based on data from 24 cadastres that had more than 1,000 registered Syrian refugees and which were matched to a city on Facebook with more than 1,000 refugee users, we observed no correlation between the number of officially registered refugees and the estimates of Facebook users. We suspect that this is due to the unclear geographic mapping between the official administrative city boundaries and the undisclosed exact geographic delineation used by Facebook.

More generally, data derived from the social media platform are not the only potential source of information on the socio-economic situation of Syrian refugees in the country. Besides official statistics, both satellite imagery as well as mobile phone metadata has seen multiple applications in recent academic literature on disaster response, migration, and socio-economic analysis: Official statistics provide reliable estimates for a range of socio-economic indicators, although often with little sub-national granularity. Earth Observation data generally captures specifics on, e.g., topography and topology at high geographical detail, even under open licenses, but does not generally fare well to differentiate complex social dynamics. Mobile metadata can picture social networks and mobility at varying degrees of spatial and temporal granularity, however, it is usually difficult to access due its proprietary and sensitive nature and as it relies on inference in most cases to capture socio-economic attributes. Facebook advertising data strikes a balance between socio-demographic scope and spatio-temporal granularity. Still, metrics derived from Facebook advertising data need to be carefully calibrated or treated otherwise to improve fairness vis-à-vis its platform-inherent biases as mentioned above. Therefore, it is not a substitute for ongoing official data collection operations such as VASyR, but a lever to break key statistics down geographically.

In order to create actual social impact, Facebook advertising could also be used as part of a multi-channel campaign for reaching out to the 56% of Syrian refugees families that are eligible for multipurpose cash assistance, but have gone unnoticed so far (according to the UNHCR's 2019 VASyR). In that regard, both targeting, campaigning, and monitoring and evaluation activities would come handily with one tool. However, prerequisites for integrating privately-held data into core processes of programme support would include reliable, beyond short-term data access arrangements, in-depth understanding of the platform-inherent biases, better coordination with other data collection operations to reduce these biases and a realistic and differentiated perspective on the specific value added of this approach.

## Data availability statement

The original contributions presented in the study are included in the article/Supplementary material, further inquiries can be directed to the corresponding author/s.

## Ethics statement

Ethical review and approval was not required for the study on human participants in accordance with the local legislation and institutional requirements. Written informed consent from the participants was not required to participate in this study in accordance with the national legislation and the institutional requirements.

## Author contributions

MF, ZV, TK, EL, AL, RA, FM, and IW contributed to conception and design of the study. MF performed the data collection. MF and IW performed the analysis. MF, ZV, TK, and IW wrote the first draft of the manuscript. EL, AL, FM, and IW oversaw partnerships and outreach. All authors contributed to manuscript revision, read, and approved the submitted version.

## Funding

This research was supported by United Nations Economic and Social Commission for Western Asia (ESCWA). EL at Universitat Pompeu Fabra is supported by funding from the European Commission's Marie Skłodowska-Curie Actions (MSCA) program via an individual fellowship. IW at Saarland University is supported by the Alexander von Humboldt Foundation under their program for establishing professorships for Artificial Intelligence^11^. We acknowledge support by the Deutsche Forschungsgemeinschaft (DFG, German Research Foundation) and Saarland University with in the funding programme Open Access Publishing.

## Conflict of interest

The authors declare that the research was conducted in the absence of any commercial or financial relationships that could be construed as a potential conflict of interest.

## Publisher's note

All claims expressed in this article are solely those of the authors and do not necessarily represent those of their affiliated organizations, or those of the publisher, the editors and the reviewers. Any product that may be evaluated in this article, or claim that may be made by its manufacturer, is not guaranteed or endorsed by the publisher.

## Footnotes

^2^https://www.bloomberg.com/news/articles/2021-09-21/lebanon-s-inflation-rises-to-highest-globally-as-crisis-deepens (accessed August 24, 2022).

^3^https://www.gdeltproject.org/ (accessed August 24, 2022).

^4^https://www.facebook.com/business/tools/ads-manager (accessed August 24, 2022).

^5^https://datareportal.com/reports/digital-2022-lebanon (accessed August 24, 2022).

^6^ https://developers.facebook.com/docs/marketing-apis/overview

^7^ https://github.com/maraujo/pySocialWatcher

^8^ https://data.unhcr.org/en/documents/details/73287

^9^ https://ialebanon.unhcr.org/vasyr/

^10^https://www.thestreet.com/investing/apple-takes-aim-at-android-users-with-iphone-se (accessed October 26, 2022).

^11^ https://www.humboldt-foundation.de/en/apply/sponsorship-programmes/alexander-von-humboldt-professorship
